# Invariant Quantum States of Quadratic Hamiltonians

**DOI:** 10.3390/e23050634

**Published:** 2021-05-19

**Authors:** Viktor V. Dodonov

**Affiliations:** 1 Institute of Physics, University of Brasilia, P.O. Box 04455, Brasilia 70919-970, DF, Brazil; vdodonov@unb.br; 2 International Center for Physics, University of Brasilia, Brasilia 70919-970, DF, Brazil

**Keywords:** covariance matrix, positively (semi)definite matrices, symplectic transformations, charged particle in homogeneous magnetic fields, generalized frequency converter

## Abstract

The problem of finding covariance matrices that remain constant in time for arbitrary multi-dimensional quadratic Hamiltonians (including those with time-dependent coefficients) is considered. General solutions are obtained.

## 1. Introduction

I consider a closed quantum system described by means of a homogeneous quadratic Hamiltonian

(1) $$H\;=\;\frac{1}{2}\sum_{j,k=1}^{2N}B_{jk}\left(t\right)q_{j}q_{k}=\frac{1}{2}\;qB\left(t\right)q.$$

Here, q is the 2N-dimensional vector, whereas *B* is a 2N×2N matrix. This vector and matrix are divided into the *N*-dimensional blocks as follows,

(2) $$q\;=\;\left[\begin{array}{c} p\\ x \end{array}\right],\;B\;=\;∥\begin{array}{cc} b_{1} & b_{2}\\ b_{3} & b_{4} \end{array}∥.$$

It is assumed that x and p are the *N*-dimensional vectors of the Cartesian coordinates and conjugated momenta, with *N* being the number of degrees of freedom of the system. The N×N matrices $b_{j}$ may be arbitrary functions of time (j=1,2,3,4). Evidently, matrix *B* can always be symmetrized; thus, I assume that $b_{1}={\tilde{b}}_{1}$, $b_{4}={\tilde{b}}_{4}$, $b_{2}={\tilde{b}}_{3}$; the tilde means the matrix transposition. If the Hamiltonian is Hermitian, then matrix *B* is real.

Many statistical properties of the quantum system are contained in the 2N×2N symmetric covariance matrix

(3) $$M\;=\;∥\bar{q_{j}q_{k}}∥=∥\begin{array}{cc} M_{pp} & M_{px}\\ M_{xp} & M_{xx} \end{array}∥,\;M_{px}\;=\;{\tilde{M}}_{xp},$$

where the (co)variances are defined as

(4) $$\bar{q_{j}q_{k}}=\frac{1}{2}\;\langle {\hat{q}}_{j}{\hat{q}}_{k}+{\hat{q}}_{k}{\hat{q}}_{j}\rangle -\langle {\hat{q}}_{j}\rangle \langle {\hat{q}}_{k}\rangle ,$$

and the splitting of matrix M in N×N blocks is performed in accordance with the structure of the 2N-dimensional vector q=(p,x). Formula (4) can be re-written as

(5) $$\frac{1}{2}\;\langle {\hat{q}}_{j}{\hat{q}}_{k}+{\hat{q}}_{k}{\hat{q}}_{j}\rangle =C_{jk}+Q_{jk},\;C_{jk}=\langle {\hat{q}}_{j}\rangle \langle {\hat{q}}_{k}\rangle ,\;Q_{jk}=\bar{q_{j}q_{k}}.$$

The remarkable property of quadratic Hamiltonians is the total independence of the first-order mean values $\langle {\hat{q}}_{j}\rangle$ and the covariances $\bar{q_{j}q_{k}}$. Moreover, the evolution of the mean values $\langle {\hat{q}}_{j}\rangle$ is governed by the *classical* equations of motion. Therefore, Formula (5) demonstrates that the average value of any symmetric bilinear combination of canonical coordinate and momentum operators is the sum of two independent parts: the classical part $C_{jk}$ and the quantum part $Q_{jk}$, which is determined by quantum fluctuations only.

If one is concerned with the *quantum* properties of the system under study, then, the main object is the covariance matrix M. Its evolution is governed by the equation

(6) $$dM/dt\;=\;MB\left(t\right)\Sigma -\Sigma B\left(t\right)M,\;\Sigma \;=\;∥\begin{array}{cc} 0 & E_{N}\\ -E_{N} & 0 \end{array}∥,$$

where $E_{N}$ is the N×N unity matrix. Therefore, matrix M depends on time in the most general case.

Recently, the authors of [1] introduced the concept of *invariant states*, i.e., quantum states with *time-independent* covariance matrices. They demonstrated the existence of such states in the case of N=2, considering an example of a frequency converter. The invariant covariance matrix must satisfy the special case of Equation (6):

(7) MB(t)Σ=ΣB(t)M.

To solve Equation (7) for the 4×4 matrix M, the authors of [1] transformed the matrix equation into an equivalent set of 10 equations for the components of a vector constructed from 10 independent elements of this symmetric matrix.

The aim of the present study is to solve Equation (7) directly in the matrix form and analyze possible forms of the invariant covariance matrices, both for the constant matrices *B* and for some specific time-dependent matrices B(t).

## 2. Simple Solutions for Time-Independent Hamiltonians

If matrix *B* does not depend on time, any time-independent solution to Equation (7) is the solution to Equation (6) as well. The simplest solution is $M_{*}=\eta B^{-1}$ with η>0. If matrix *B* is positively definite (as happens for physical Hamiltonians), then matrix $M_{*}$ is positively definite automatically. However, physical covariance matrices must be not only positively definite, but they must satisfy a more strong restriction of positive definiteness of matrix Y=M+iℏΣ/2 [2,3]. In particular, the following inequality must be satisfied:

(8) $$\det M\geq {\left(\hbar /2\right)}^{2N}.$$

Therefore, the coefficient η must satisfy the inequalty $\eta \geq {\left(\det B\right)}^{1/2N}\hbar /2$. For example, considering the one-dimensional harmonic oscillator with the diagonal matrix $B=diag(m^{-1},m\omega^{2})$, we have $\det B=\omega^{2}$, so that the minimal choice $\eta_{min}=\hbar \omega /2$ yields the covariance matrix of the vacuum state. The value $\eta_{n}=\left(2n+1\right)\eta_{min}$ corresponds to the covariance matrix of the *n*-th energy eigenstate.

One can easily check that any matrix ${\left(\Sigma B\right)}^{k}\Sigma$ (with integer *k*) also satisfies Equation (7). Due to the linearity of this equation, one can write a more general solution as

(9) $$M=\sum_{k=\pm 1,\pm 3,\ldots }c_{k}{\left(\Sigma B\right)}^{k}\Sigma ,$$

where the coefficients $c_{k}$ must be chosen in such a way that matrix Y is positively definite. The absence of even powers of matrix ΣB in the sum (series) (9) is explained by the fact that all matrices ${\left(\Sigma B\right)}^{2k}\Sigma$ are antisymmetric.

It is known that the covariance matrix does not depend on time for any quantum mixture of energy eigenstates, in particular, for the equilibrium states. For such states, we have [3,4]

(10) $$M_{eq}=\left(\hbar /2\right)\cot\left(\hbar \beta \Sigma B/2\right)\Sigma ,$$

where β is the inverse temperature parameter. Formula (10) is the consequence of the general formula for the Gaussian Wigner function of homogeneous quadratic systems [3,5,6,7]

(11) $$W_{Gauss}\left(q\right)∼\exp\left(-\frac{1}{2}qM^{-1}q\right)$$

and the explicit expression for the equilibrium Wigner function of the most general homogeneous quadratic Hamiltonians [8,9]

(12) $$W_{eq}\left(q\right)∼\exp\left[\hbar^{-1}q\Sigma \tan\left(\hbar \beta \Sigma B/2\right)q\right].$$

Formula (10) has the form of (9) with k≥−1.

Matrix ${\left(\Sigma B\right)}^{2}$ has the following block form:

$${\left(\Sigma B\right)}^{2}\;=\;∥\begin{array}{cc} {\tilde{b}}_{2}^{2}-b_{4}b_{1} & {\tilde{b}}_{2}b_{4}-b_{4}b_{2}\\ b_{2}b_{1}-b_{1}{\tilde{b}}_{2} & b_{2}^{2}-b_{1}b_{4} \end{array}∥.$$

This matrix is proportional to the unit matrix if N=1, when all blocks are usual numbers. Consequently, the sum (series) (9) is proportional to $B^{-1}$ for all one-dimensional quadratic systems with non-degenerate matrix *B*. This means that one-dimensional systems possess a unique set of invariant covariance matrices, with the form $\eta B^{-1}$ (or an equivalent form ξΣBΣ with ξ<0 in order to maintain the positive definiteness). This result can be easily checked by directly solving Equation (7) when all matrices have the dimension 2×2. In particular, any Gaussian invariant state can be considered as a thermal state with some effective temperature in the case of N=1.

### Two-Dimensional Examples: A Charged Oscillator and a Charge in a Homogeneous Magnetic Field

However, the situation can be different for multidimensional systems. As an example of a two-dimensional system, let us consider an isotropic charged two-dimensional oscillator in the plane xy, placed in a homogeneous magnetic field H perpendicular to this plane. For the circular gauge of the vector potential, A=H(−y,x,0)/2, the Hamiltonian has the form (using dimensionless variables)

(13) $$\hat{H}=\frac{1}{2}{\left({\hat{p}}_{x}+\omega \hat{y}\right)}^{2}+\frac{1}{2}{\left({\hat{p}}_{y}-\omega \hat{x}\right)}^{2}+\frac{1}{2}g\left({\hat{x}}^{2}+{\hat{y}}^{2}\right),$$

so that the 2×2 blocks of 4×4 matrix *B* can be written in the form

$$b_{1}=E_{2},\;b_{2}=-{\tilde{b}}_{2}=\omega \Sigma_{2},\;b_{4}=-b_{2}^{2}+g^{2}E_{2}=\left(\omega^{2}+g^{2}\right)E_{2},$$

where ω is the Larmor frequency and *g* is the oscillator frequency. Using the Frobenius formula for the inversion of block matrices [10],

$${∥\begin{array}{cc} a & b\\ c & d \end{array}∥}^{-1}\;=\;∥\begin{array}{cc} a^{-1}+a^{-1}bh^{-1}ca^{-1} & -a^{-1}bh^{-1}\\ -h^{-1}ca^{-1} & h^{-1} \end{array}∥,\;h=d-ca^{-1}b,$$

one can obtain the following *independent* solutions to Equation (7) (containing the matrix $b_{2}$ with different signs):

(14) $$M_{1}=g^{2}B^{-1}\;=\;∥\begin{array}{cc} \left(\omega^{2}+g^{2}\right)E_{2} & -\omega \Sigma_{2}\\ \omega \Sigma_{2} & E_{2} \end{array}∥,\;M_{2}\;=\;-\Sigma B\Sigma =∥\begin{array}{cc} \left(\omega^{2}+g^{2}\right)E_{2} & \omega \Sigma_{2}\\ -\omega \Sigma_{2} & E_{2} \end{array}∥.$$

These matrices should be multiplied by some factors in order to obtain positively definite matrices satisfying the condition detM≥1/16, according to Equation (8). The determinants of the matrices $M_{1,2}$ can be calculated with the aid of the Frobenius determinant formulas

$$\det∥\begin{array}{cc} a & b\\ c & d \end{array}∥\;=\;\det\left(a\right)\det\left(d-ca^{-1}b\right)\;=\;\det\left(d\right)\det\left(a-bd^{-1}c\right).$$

Therefore, $\det\left(M_{1}\right)=\det\left(M_{2}\right)=\det\left(B\right)=g^{4}$. Consequently, matrices (14) cannot be used directly in the limit case of g=0 (a free charged particle in a homogeneous magnetic field). However, in this case, one can use linear combinations of $M_{1}$ and $M_{2}$ in the form (assuming ω>0)

(15) $$M_{\gamma }=\frac{N}{2\sqrt{1-\gamma^{2}}}∥\begin{array}{cc} \omega E_{2} & \gamma \Sigma_{2}\\ -\gamma \Sigma_{2} & \omega^{-1}E_{2} \end{array}∥,\;\left|\gamma \right|<1,\;\det\left(M_{\gamma }\right)\;=\;N^{4}/16.$$

The condition $\det\left(M_{\gamma }\right)\geq 1/16$ is satisfied if N≥1. However, this is only a necessary condition of positivity of matrix Y=M+iℏΣ/2. One has to also check the positivity of all principal minors of matrix Y. However, in the case under study, one can use another way that sheds more light on the physical aspects of the problem.

It is known (at least since the paper [11]) that the following set of linear combinations of the canonical coordinates and momenta is very useful for the description of the motion of a free charged particle (of unit mass and charge) in the homogeneous magnetic field:

(16) $${\hat{x}}_{c}=\frac{1}{2}\left(\hat{x}+{\hat{p}}_{y}/\omega \right),\;{\hat{y}}_{c}=\frac{1}{2}\left(\hat{y}-{\hat{p}}_{x}/\omega \right),$$

(17) $${\hat{x}}_{r}=\hat{x}-{\hat{x}}_{c}=\frac{1}{2}\left(\hat{x}-{\hat{p}}_{y}/\omega \right),\;{\hat{y}}_{r}=\hat{y}-{\hat{y}}_{c}=\frac{1}{2}\left(\hat{y}+{\hat{p}}_{x}/\omega \right).$$

The first pair describes coordinates of the center of a circle where the particle rotates around, whereas the second pair of observables consists of two relative coordinates with respect to this center. The vectors q and $z=(x_{r},y_{r},x_{c},y_{c})$ are related by means of the linear transformation z=Uq with

$$U\;=\;\frac{1}{2}∥\begin{array}{cc} -\omega^{-1}\Sigma_{2} & E_{2}\\ \omega^{-1}\Sigma_{2} & E_{2} \end{array}∥,\;U^{-1}\;=\;∥\begin{array}{cc} \omega \Sigma_{2} & -\omega \Sigma_{2}\\ E_{2} & E_{2} \end{array}∥.$$

Hence, the covariance matrix Z with respect to the components of vector z has the form

(18) $$Z_{\gamma }=UM_{\gamma }\tilde{U}=\frac{N}{4\omega }∥\begin{array}{cc} E_{2}\sqrt{\frac{1+\gamma }{1-\gamma }} & 0\\ 0 & E_{2}\sqrt{\frac{1-\gamma }{1+\gamma }} \end{array}∥.$$

Due to the commutation relations

(19) $$\left[{\hat{x}}_{r},{\hat{y}}_{r}\right]=\left[{\hat{y}}_{c},{\hat{x}}_{c}\right]=i/\left(2\omega \right),$$

we have the uncertainty inequalities

(20) $$\sigma_{x_{r}}\sigma_{y_{r}}\geq {\left(4\omega \right)}^{-2},\;\sigma_{x_{c}}\sigma_{y_{c}}\geq {\left(4\omega \right)}^{-2}.$$

Consequently, comparing Equations (18) and (20), one arrives at a more strong restriction

$$N^{2}\geq \left(1+|\gamma |\right)/\left(1-|\gamma |\right).$$

It is known that the covariance matrix determines the quantum state uniquely (up to an insignificant phase) if the state is *Gaussian*. In this case, the state is pure provided detM=1. Therefore, the only pure invariant Gaussian state of a charged particle in a homogeneous magnetic field has the covariance matrix (15) or (18) with γ=0. This is nothing but the *coherent state* introduced by Malkin and Man’ko in 1968 [12].

Now, let us note that the Hamiltonian (13) with g=0 can be rewritten in therms of the relative coordinates only:

(21) $$\hat{H}=2\omega^{2}\left({\hat{x}}_{r}^{2}+{\hat{y}}_{r}^{2}\right).$$

Since the pairs $\left({\hat{x}}_{r},{\hat{y}}_{r}\right)$ and $\left({\hat{x}}_{c},{\hat{y}}_{c}\right)$ are totally independent, the most general invariant 4×4 covariance matrix for the Hamiltonian (21) has the form

(22) $$Z_{gen}\;=\;∥\begin{array}{cc} \eta E_{2} & 0\\ 0 & C \end{array}∥,\;\eta \geq {\left(4\omega \right)}^{-1},\;C\;=\;∥\begin{array}{cc} c_{1} & c_{12}\\ c_{12} & c_{2} \end{array}∥,\;c_{1,2}>0,\;c_{1}c_{2}-c_{12}^{2}\geq {\left(4\omega \right)}^{-2}.$$

Returning to the q-variables, one can obtain the following most general invariant matrix:

(23) $$M_{gen}=U^{-1}Z_{gen}{\tilde{U}}^{-1}\;=\;∥\begin{array}{cc} \omega^{2}G & \omega K\\ \omega \tilde{K} & F \end{array}∥,$$

$$G\;=\;∥\begin{array}{cc} \eta +c_{2} & -c_{12}\\ -c_{12} & \eta +c_{1} \end{array}∥,\;F\;=\;∥\begin{array}{cc} \eta +c_{1} & c_{12}\\ c_{12} & \eta +c_{2} \end{array}∥,\;K\;=\;∥\begin{array}{cc} -c_{12} & \eta -c_{2}\\ c_{1}-\eta & -c_{12} \end{array}∥.$$

Therefore, the most general invariant matrix is determined by three positive parameters ($\eta ,c_{1},c_{2}$) and one parameter ($c_{12}$) of an arbitrary sign. However, all these parameters must obey certain restrictions, given in Equation (22).

## 3. General Solutions for Positive Time-Independent Hamiltonians

The results of the preceding subsection show the general way to solve the problem in the case of an arbitrary *positively definite* time-independent matrix *B*. It is well known (since the paper [13], whose results can be found also in book [14]; see also [15,16,17,18,19]) that such matrices can be diagonalized by means of *symplectic* (canonical) transformations of the form

(24) $$TB\tilde{T}=B_{*}=diag\left(B_{1},B_{2},\ldots ,B_{N}\right),\;T\Sigma \tilde{T}=\Sigma ,\;B_{j}=diag\left(\mu_{j},\nu_{j}\right),\;\mu_{j}>0,\;\nu_{j}>0.$$

Putting $B=T^{-1}B_{*}{\tilde{T}}^{-1}$ in Equation (7), one obtains an equivalent equation

$$M_{*}B_{*}\Sigma =\Sigma B_{*}M_{*},\;M_{*}={\tilde{T}}^{-1}MT^{-1}.$$

Using the results of the preceding section related to the case of N=1, one can write the general solution:

(25) $$M=\tilde{T}diag\left(\eta_{1}B_{1}^{-1},\eta_{2}B_{2}^{-1},\ldots ,\eta_{N}B_{N}^{-1}\right)T,\;\eta_{j}\geq \hbar \sqrt{\mu_{j}\nu_{j}}/2.$$

This depends on *N* positive parameters $\eta_{j}$. However, the block matrices $B_{j}$ are not determined uniquely: only the products $\mu_{j}\nu_{j}$ are fixed by the eigenvalues of matrix *B*, while the ratio $\mu_{j}/\nu_{j}$ can be considered as an additional parameter. Therefore, the total number of parameters is 2N for N≥2.

## 4. Specific Time-Dependent Hamiltonians Admitting Invariant Covariance Matrices

Equation (7) has an obvious solution $M=\eta E_{2N}$ for any matrix $B_{0}\left(t\right)$ satisfying the condition $B_{0}\Sigma =\Sigma B_{0}$. This results in the following relations between the N×N blocks:

(26) $$b_{1}=b_{4},\;{\tilde{b}}_{2}=-b_{2}.$$

Multiplying both sides of the equality $B_{0}\Sigma =\Sigma B_{0}$ by some *symplectic* time-independent matrix Λ from the left and matrix $\tilde{\Lambda }$ from the right, we find the following general set of solutions to Equation (7):

(27) $$M=\eta \Lambda \tilde{\Lambda },\;B\left(t\right)={\tilde{\Lambda }}^{-1}B_{0}\left(t\right)\Lambda^{-1}.$$

Remember that symplectic matrices obey the relation

(28) $$\Lambda \Sigma \tilde{\Lambda }=\Sigma .$$

The consequence is |detΛ|=1. Hence, η≥ℏ/2. Clearly, matrix $\Lambda \tilde{\Lambda }$ is positively definite. Another consequence of the identity (28) is the formula $\Lambda^{-1}=-\Sigma \tilde{\Lambda }\Sigma$. Therefore,

(29) $$B\left(t\right)=\Sigma \Lambda \Sigma B_{0}\left(t\right)\Sigma \tilde{\Lambda }\Sigma .$$

Finally, we obtain the following expressions for 2N×2N matrices in terms of N×N blocks:

(30) $$\Lambda \tilde{\Lambda }=∥\begin{array}{cc} \lambda_{1} & \lambda_{2}\\ \lambda_{3} & \lambda_{4} \end{array}∥\cdot ∥\begin{array}{cc} {\tilde{\lambda }}_{1} & {\tilde{\lambda }}_{3}\\ {\tilde{\lambda }}_{2} & {\tilde{\lambda }}_{4} \end{array}∥=∥\begin{array}{cc} \lambda_{1}{\tilde{\lambda }}_{1}+\lambda_{2}{\tilde{\lambda }}_{2} & \lambda_{1}{\tilde{\lambda }}_{3}+\lambda_{2}{\tilde{\lambda }}_{4}\\ \lambda_{3}{\tilde{\lambda }}_{1}+\lambda_{4}{\tilde{\lambda }}_{2} & \lambda_{3}{\tilde{\lambda }}_{3}+\lambda_{4}{\tilde{\lambda }}_{4} \end{array}∥,$$

(31) $$B\left(t\right)=∥\begin{array}{cc} \lambda_{4}\alpha {\tilde{\lambda }}_{4}+\lambda_{3}\alpha {\tilde{\lambda }}_{3}+\lambda_{3}\beta {\tilde{\lambda }}_{4}-\lambda_{4}\beta {\tilde{\lambda }}_{3} & -\lambda_{4}\alpha {\tilde{\lambda }}_{2}-\lambda_{3}\alpha {\tilde{\lambda }}_{1}+\lambda_{4}\beta {\tilde{\lambda }}_{1}-\lambda_{3}\beta {\tilde{\lambda }}_{2}\\ -\lambda_{2}\alpha {\tilde{\lambda }}_{4}-\lambda_{1}\alpha {\tilde{\lambda }}_{3}+\lambda_{2}\beta {\tilde{\lambda }}_{3}-\lambda_{1}\beta {\tilde{\lambda }}_{4} & \lambda_{2}\alpha {\tilde{\lambda }}_{2}+\lambda_{1}\alpha {\tilde{\lambda }}_{1}+\lambda_{1}\beta {\tilde{\lambda }}_{2}-\lambda_{2}\beta {\tilde{\lambda }}_{1} \end{array}∥,$$

where $\alpha \left(t\right)=\tilde{\alpha }\left(t\right)$ and $\beta \left(t\right)=-\tilde{\beta }\left(t\right)$ can be arbitrary symmetric and antisymmetric N×N matrices. Blocks $\lambda_{j}$ of a symplectic matrix Λ satisfy many identities [6]:

(32) $$\lambda_{1}{\tilde{\lambda }}_{2}=\lambda_{2}{\tilde{\lambda }}_{1},\;\lambda_{3}{\tilde{\lambda }}_{4}=\lambda_{4}{\tilde{\lambda }}_{3},\;{\tilde{\lambda }}_{1}\lambda_{3}={\tilde{\lambda }}_{3}\lambda_{1},\;{\tilde{\lambda }}_{4}\lambda_{2}={\tilde{\lambda }}_{2}\lambda_{4},$$

(33) $$\lambda_{4}{\tilde{\lambda }}_{1}-\lambda_{3}{\tilde{\lambda }}_{2}=\lambda_{1}{\tilde{\lambda }}_{4}-\lambda_{2}{\tilde{\lambda }}_{3}=E_{N},\;{\tilde{\lambda }}_{4}\lambda_{1}-{\tilde{\lambda }}_{2}\lambda_{3}={\tilde{\lambda }}_{1}\lambda_{4}-{\tilde{\lambda }}_{3}\lambda_{2}=E_{N}.$$

The case of N=1 is trivial and non-interesting because β(t)=0 and $B_{0}∼E_{2}$. However, the situation can be non-trivial for N≥2. For example, we have the following set of possible matrices α(t) and β(t) for N=2:

(34) $$\alpha \left(t\right)=∥\begin{array}{cc} r(t) & g(t)\\ g(t) & s(t) \end{array}∥,\;\beta \left(t\right)=∥\begin{array}{cc} 0 & f(t)\\ -f(t) & 0 \end{array}∥.$$

Consequently, the invariant matrix $M=\eta E_{2N}$ exists for any time-dependent Hamiltonian of the form

(35) $$\hat{H}=\frac{1}{2}\left[r\left(t\right)\left({\hat{p}}_{x}^{2}+{\hat{x}}^{2}\right)+s\left(t\right)\left({\hat{p}}_{y}^{2}+{\hat{y}}^{2}\right)+2g\left(t\right)\left({\hat{p}}_{x}{\hat{p}}_{y}+\hat{x}\hat{y}\right)+2f\left(t\right)\left({\hat{p}}_{x}\hat{y}-\hat{x}{\hat{p}}_{y}\right)\right].$$

Introducing the standard bosonic annihilation and creation operators,

$$\hat{a}=\frac{\hat{x}+i{\hat{p}}_{x}}{\sqrt{2}},\;{\hat{a}}^{†}=\frac{\hat{x}-i{\hat{p}}_{x}}{\sqrt{2}},\;\hat{b}=\frac{\hat{y}+i{\hat{p}}_{y}}{\sqrt{2}},\;{\hat{b}}^{†}=\frac{\hat{y}-i{\hat{p}}_{y}}{\sqrt{2}},$$

one can re-write Hamiltonian (35) as

(36) $$\hat{H}=r\left(t\right)\left({\hat{a}}^{†}\hat{a}+1/2\right)+s\left(t\right)\left({\hat{b}}^{†}\hat{b}+1/2\right)+\xi \left(t\right)\hat{a}{\hat{b}}^{†}+\xi^{*}\left(t\right)\hat{b}{\hat{a}}^{†},\;\xi \left(t\right)=g\left(t\right)+if\left(t\right).$$

This is a generalization of the frequency converter Hamiltonian considered in paper [1] with r,s=const and $\xi \left(t\right)=\kappa e^{i\omega t}$. Further generalizations can be obtained by means of transformation (29). For example, using the simplest symplectic transformation with diagonal matrices $\lambda_{1}=\lambda_{4}^{-1}$ and $\lambda_{2}=\lambda_{3}=0$, one can introduce different masses and frequencies in the basic Hamiltonian (35). Then, the invariant matrix M will remain diagonal, but with different elements.

## 5. Discussion

The main result of this paper is the description of a general structure of the covariance matrices that do not depend on time for quadratic *N*-dimensional Hamiltonians. If the *time independent* Hamiltonian matrix *B* (of dimension 2N×2N) is positively definite, such invariant matrices depend on 2N parameters for N≥2 (and a single parameter if N=1). If the symmetric matrix *B* is only non-negatively definite, the situation depends on the degeneracy of zero eigenvalues of this matrix: the invariant matrices exist if the number of such eigenvalues is even (an example is a free charge moving in a homogeneous magnetic field as considered in Section 2); however, these do not exist if this number is odd (an example is the free particle Hamiltonian $\hat{H}={\hat{p}}^{2}/\left(2m\right)$).

A general structure of *time-dependent* quadratic Hamiltonians admitting invariant covariance matrices has been established. Such Hamiltonians are generalizations of the frequency converter Hamiltonians of quantum optics, considered in paper [1].

A few words regarding the place of the invariant states considered in this paper in the world of other quantum invariants appears relevant. If the quadratic Hamiltonian does not depend on time then there exist some specific linear combinations of a few second-order moments that do not depend on time: well known examples are the mean value of the energy (and angular momentum in the case of additional symmetries). Moreover, such kinds of specific linear combinations can be constructed for arbitrary time-dependent quadratic Hamiltonians, as was shown by Lewis and Riesenfeld [20]. Their method of time-dependent quantum operators and integrals of motion was generalized and further developed by Malkin and Man’ko with collaborators [6,21,22,23,24,25,26,27,28,29,30,31] and other authors [32,33,34,35,36,37,38,39,40,41,42,43,44,45,46,47,48,49,50,51,52,53,54,55,56].

The important applications of the Lewis–Riesenfeld and Malkin–Man’ko invariants include, e.g., the “inverse engineering” of quadratic Hamiltonians: a search of “shortcuts to adiabaticity” in different kinds of traps [57,58,59,60]. Other applications are related to the theory of the geometric (Berry) phase [61,62,63,64,65,66,67], invariants of non-Hermitian Hamiltonians [68,69,70], and open quantum systems [71,72,73,74].

Basic elements of the Malkin–Man’ko construction for Hamiltonian (1) are 2N linear operator integrals of motion, combined in the vector $\hat{Q}\left(t\right)=\Lambda \left(t\right)\hat{q}$, where the 2N×2N symplectic matrix Λ(t) satisfies the equation dΛ/dt=ΛΣB(t). Then, the mean value of any quadratic form $\hat{Q}\left(t\right)G\hat{Q}\left(t\right)=\hat{q}\tilde{\Lambda }\left(t\right)G\Lambda \left(t\right)\hat{q}$ with symmetric matrix *G* does not depend on time. All such quadratic invariants are linear combinations of the second-order moments with time-dependent coefficients determined by the Hamiltonian matrix *B*.

In addition to these invariants (whose values depend on matrix *B* and the initial quantum state), there exist other combinations of the second-order moments (not linear, but bilinear or multilinear) that depend on the initial state and the antisymmetric commutator matrix (Σ in the case of standard coordinates and momenta operators) but do not depend on the concrete matrix *B*. Such combinations were named *universal quantum invariants* in [3,6,75,76]. The simplest examples are “trace universal invariants” $L_{2m}=Tr\left[{\left(M\Sigma^{-1}\right)}^{2m}\right]$ with m=1,2,…,N. They obey the generalized uncertainty relations [77] ${\left(-1\right)}^{m}L_{2m}\geq N/2^{2m-1}$. Similar invariants and their special cases in the physics of particle and optical beams were considered, e.g., in the papers [78,79,80,81,82,83,84,85,86,87,88,89,90,91,92]. Such constructions are frequently used in quantum information theory under the name “symplectic invariants” [9,93,94].

The invariants mentioned above contain only *some* elements of the covariance matrix M. Moreover, each of these elements can depend on time, and only their specific combinations are time-independent. On the contrary, *all* elements of the invariant matrices studied in this paper do not depend on time. This is a novelty suggested in [1]. What are invariant quantum *states*? For the Gaussian states, there exists the direct and unique relation between the covariance matrix and the state, given by Formula (11). In this case, the majority of invariant states is represented by *quantum mixtures*.

Pure invariant states are the *vacuum* (minimum energy) states (or coherent states, as soon as the displacement operator does not change the covariance matrix), although the situation may be more complicated for certain Hamiltonians (such as those including the magnetic field). A description of invariant *non-Gaussian* pure states could be an interesting avenue of future research. Another potentially interesting problem could be the search of invariant states for *open* quantum systems, when Equation (6) is replaced with a more general equation $dM/dt=MA+\tilde{A}M+D$, where *D* is a symmetric diffusion matrix and *A* is a “drift” matrix containing the terms responsible for dissipation [3,95]. These subjects, however, require separate studies.

## Data Availability

Not applicable.
